# Root resorption of self-ligating and conventional preadjusted brackets in severe anterior crowding Class I patients: a longitudinal retrospective study

**DOI:** 10.1186/s12903-015-0100-0

**Published:** 2015-10-01

**Authors:** Weiting Chen, Abdul Azeem Aminul Haq, Yu Zhou

**Affiliations:** Department of Orthodontics, School & Hospital of Stomatology, Wenzhou Medical University, Wenzhou, Zhejiang China; School of international studies, Wenzhou Medical University, Wenzhou, Zhejiang China

**Keywords:** Self-ligating brackets, Conventional brackets, EARR

## Abstract

**Background:**

To test the null hypothesis that there is no difference in the apical root resorption seen after orthodontic treatment with the conventional brackets and the self-ligating brackets.

**Methods:**

Pre-treatment and post-treatment periapical radio-graphs of 70 patients, (35 treated with the Damon3 0.022” bracket and 35 with the 0.022” 3 M bracket) were studied. The long cone paralleling technique was used for all the radio-graphs. Any image distortion between the pre-treatment and post-treatment radio-graph was calculated and compensated for by using the crown length measurements, on the assumption that the crown length remains unaltered during the treatment period. Quantitative measurements of crown and root lengths for the maxillary and the mandibular central and lateral incisors were compared. Means and standard deviations for the percentage root resorption per tooth group were calculated. A paired *t*-test and non paired *t*-test analysis was performed to determine whether there was an appliance, treatment time, or initial age effect on the amount of root resorption seen after treatment.

**Result:**

No statistically significant difference in root resorption between the two appliance systems was found. The patient’s degree of root resorption were graded as grade 1 and grade 2 in the self-ligating group which is more than the conventional group.

**Conclusions:**

There was no significant difference in root resorption between self-ligating brackets and conventional brackets in severe crowding incisors subjects.

## Background

External apical root resorption during orthodontic treatment, referred to as ‘surface root resorption’ by Andreasen [1], is considered an unwanted consequence of orthodontic treatment that can cause biological changes in the cementum and periodontal ligament, resulting in root resorption [2, 3]. The teeth more susceptible to EARR are the maxillary and mandibular incisors, and especially the maxillary lateral incisors [4–7].

External apical root resorption (EARR) presents with a multi-factorial etiology, individual predisposition being one of the factors [8–10]. Since the greatest cause of root resorption in the population refers to orthodontic movement, a correlation exists between severity of the malocclusion and the degree of consequent root resorption [9–11]. This occurs as a result of the mechanical resources demanded and is due to long-lasting treatment [5]. In addition, characteristics that are inherent to orthodontic treatment, such as type of brackets, the mechanics used, and the type and magnitude of the forces applied [9, 10, 12], are also relevant.

Few studies have dealt with the effects of mechano-therapy on EARR. Standard edgewise, straight-wire, and Begg appliances, as well as various mechano-therapeutic approaches have been investigated [13–17]. The contributing role of continuous tooth movement across various paths, an undesirable effect known as “tooth jiggling,” and the application of inter-maxillary elastics in the development of EARR have been highlighted [18]. An interesting recent study also indicated that more EARR occurred with nickel-titanium wires and with stainless steel arch-wires [19].

The introduction of self-ligating brackets provoked the investigation of arch-wire ligation on EARR. One of the first reports on the subject was by Blake et al. [20], who tested the hypothesis that an active self-ligating bracket with an active clip might induce more EARR; their findings, however, did not confirm that hypothesis. The introduction of passive self-ligating systems, with no active spring and alignment performed by wires engaged in a passive tube, with more play, jiggling, and less friction, raises again the question of their effect on EARR. In the orthodontic literature, few studies have explored the effect on EARR of passive self-ligating systems.

Therefore, the hypothesis we tested was that the differences in the mode of wire ligation between conventional and passive self-ligating brackets affect the rate of root resorption. Our purpose was to compare the extent of root resorption of the maxillary and mandibular incisors in severe anterior crowding class I patients between self-ligating and conventional pre-adjusted brackets.

## Methods

This study was designed as a longitudinal retrospective study. The study was approved by the Ethics Committee of Wenzhou medical college (182/April 2012). Inclusion criteria were: patients presented Angle Class I malocclusion, with anterior crowding more than 6 mm. Only patients that had undergone four premolar extractions were selected. Exclusion criteria were: subjects with root resorption, Class II elastics, endodontic treatment, a history of trauma impacted canines, previous orthodontic treatment or with signs of EARR observed at the first examination, or dental anomalies of a number of teeth before treatment were excluded. Those with incomplete orthodontic records and poor quality radio-graphs were also not included.

Based on a retrospective power analysis, a total of 66 patients were required to demonstrate a clinically meaningful difference of 0.85 mm in root resorption between the appliance systems with 0.05 significance level and a power of 80 %.

**Fig 1:**
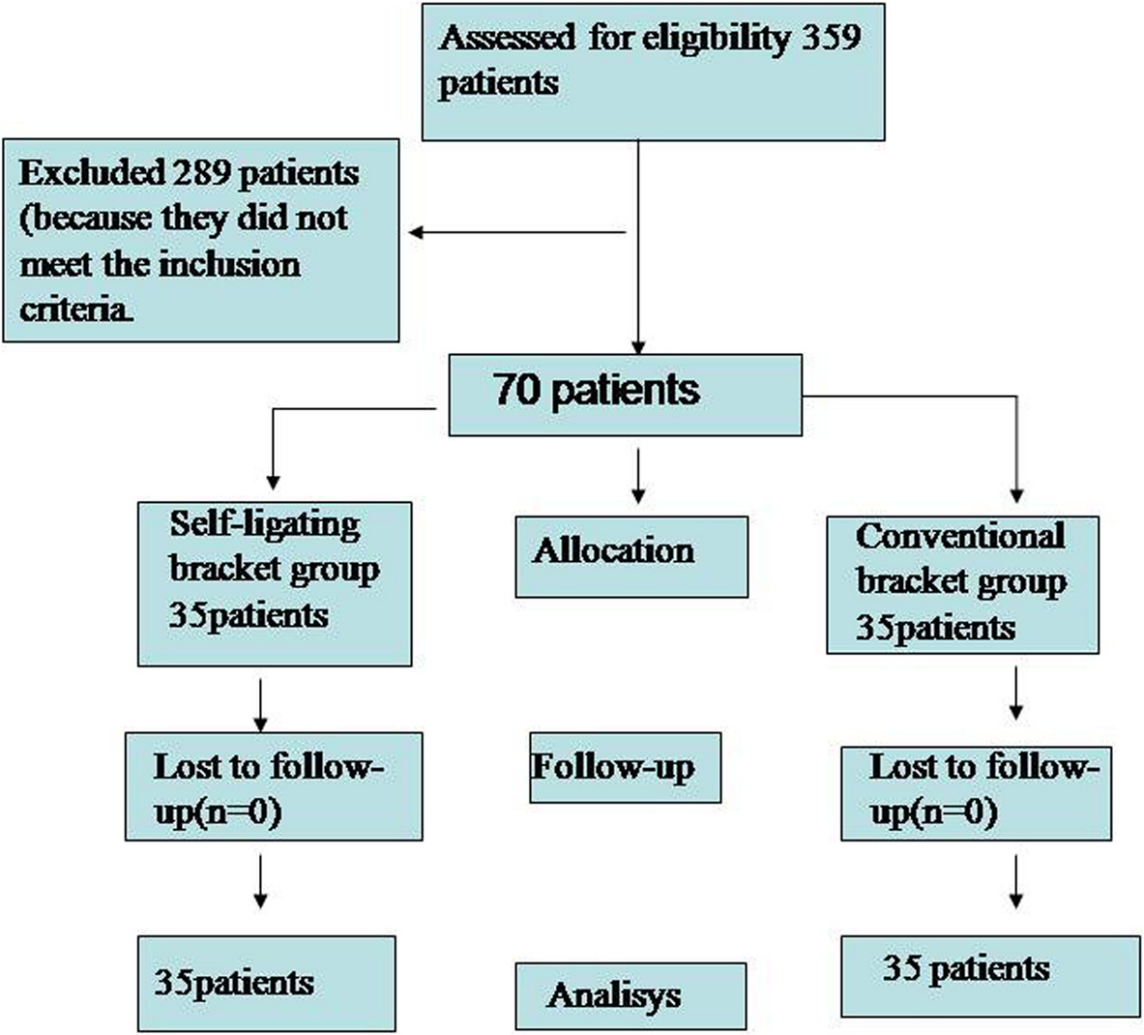
Flow diagram of participants through each stage of the trial

For this retrospective study, 359 participants were screened in the Department of orthodontic, hospital of stomatology, Wenzhou Medical University. 289 participants were excluded because they did not meet the inclusion criteria. At last, 70 patients were enrolled in this study, for further details see Fig. 1. They were divided into two groups: Group I (*n* = 35; 18 female and 17 male subjects using passive self-ligating brackets with a 0.022 × 0.028 in. slot (Damon 3, OMRCO, USA) and Group II (*n* = 35; 19 female and 16 male subjects using conventional pre-adjusted brackets with a 0.022 × 0.028 in. slot (3 M Unitek, California, USA).

Orthodontic mechanics was used a wire sequence characterized by an initial 0.012 in. nitinol or a 0.014 in. nitinol, followed by 0.016, 0.018, 0.019 × 0.025 in. nitinol, and 0.019 × 0.025 in. stainless steel arch-wires (3 M Unitek, California, USA). Slide mechanics to closed space. Trans-palatal arch or nance arch were used to enhance anchorage.

Standardized periapical radio-graphs were obtained by a single operator with the long-cone paralleling technique (SIEMENS, SIDEXIS XG, Germany) . The radio-graphs were developed with SIDEXIS automatic dental film processor . The measures were performed to the nearest 0.01 mm, using the image analysis system (SIEMENS, SIDEXIS XG, Germany). Lengths of the maxillary and mandibular incisors were measured on intra-oral periapical films before and after active treatment. Root length was measured from the CEJ to the apex. All measurements were obtained by perpendicularly projecting these points on the long axis of the tooth, which followed as accurately as possible the root canal. The most distinct CEJ landmark, either mesial or distal, was used, but once decided, the same side was used both for the pre- and post-treatment radio-graphs.

Any image distortion between the pre-treatment and post-treatment radio-graphic exposures was calculated using the crown length registrations. This method was described by Linge and Linge [4]. A correction factor was calculated to relate the pre-treatment and post-treatment radio-graphs.

$$ \mathrm{C}\mathrm{orrection}\ \mathrm{F}\mathrm{actor}\ \left(\mathrm{C}\mathrm{F}\right) = \mathrm{C}1/\mathrm{C}2 $$

Where: C1 = Crown length on pre-treatment radio-graph.

C2 = Crown length on post-treatment radio-graph.

The apical root resorption per tooth was calculated as follow:

$$ \mathrm{Apical}\ \mathrm{root}\ \mathrm{resorption}\ \left(\mathrm{A}\mathrm{R}\mathrm{R}\right) = \mathrm{R}\ 1 - \left(\mathrm{R}\ 2 \times \mathrm{C}\mathrm{F}\right) $$

Where: R 1 = Root length before treatment.

R 2 = Root length after treatment.

It was decided to express the root resorption seen as the percentage shortening per tooth. This percent value is a better comparative value since the differences in the root lengths of various teeth, make comparisons of root resorption values in millimeters more difficult.

**Fig 2:**
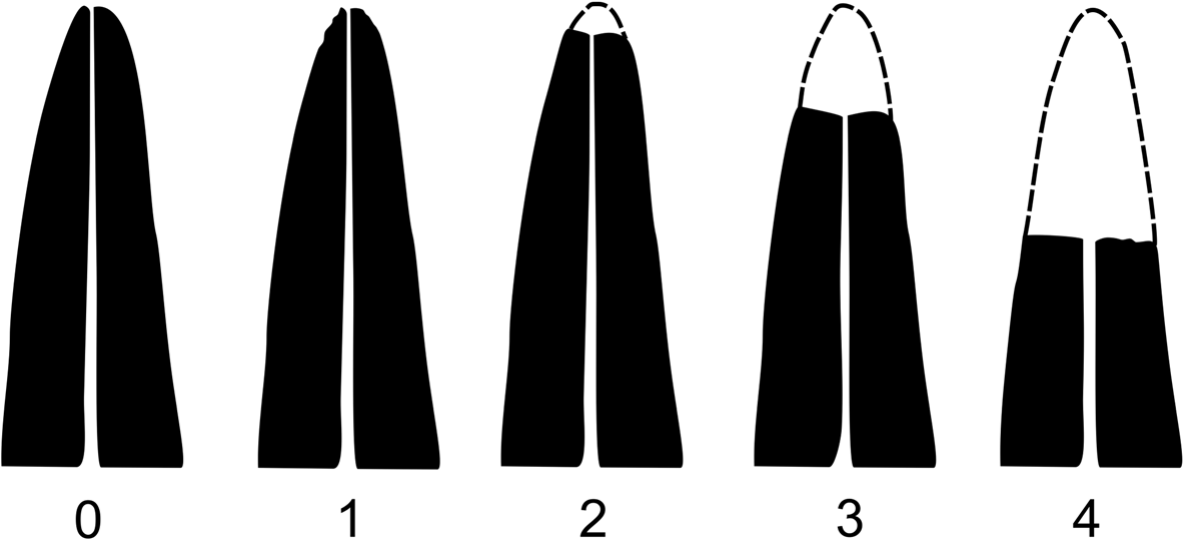
Scoring system

Root resorption evaluation was blindly performed by one author on the final periapical radio-graphs and initial radio-graphs. The method of Malmgren [21] was used to evaluate the severity of apical root resorption, ranking it into 5° (Fig. 2). Four scores, corresponding to each anterior incisor, for each patient were obtained, a total of 280 scores per group.

### Error study

Twenty randomly selected patients, 10 from each group, had the amount of any root resorption re-evaluated and their radio-graphs were retraced and remeasured by the same examiner after one month. For root resorption evaluation, intra-examiner agreement was calculated with Kappa statistics and Dahlberg formula (casual errors).

### Statistical analyses

Chi-square was used to show the distribution of the teeth among the scores of root resorption according to the method of Malmgren [22].

A paired *t*-test was employed to compare the degree of root resorption in each group between T1 and T2 periods, and a non-paired *t*-test was used for comparison between both groups. In all statistical tests, the significance level was set at 5 %. Statistical calculations were made with Statistic software SPSS1.0 (version 20.0, IBM Inc, USA).

## Results

Kappa statistics showed almost perfect agreement between the first and second root resorption intra-examiner evaluation. There were no statistically significant systematic errors and the casual errors were within acceptable limits (*P* =0.656 and Dahlberg = 0.27).

The groups were matched regarding the initial ages, treatment time. No statistically significant difference was found in the comparison of the initial ages and treatment time between the two groups (Table 1).

**Table 1:** Comparison for initial ages and treatment time between Group I (Self-ligating Brackets) and Group II (Conventional Preadjusted Brackets)

Variable	Group 1		Group 2		*P*
	Mean	SD	Mean	SD	
Initial age (years)	13.52	2.84	13.42	2.50	NS
Treatment time	20.53	3.62	20.34	3.40	NS

NS mean: No Statistically significant difference (*P* >05)

A statistically significant difference occurred in all teeth in the comparison between T1 and T2 for patients of Group I (Table 2).

**Table 2:** Comparison of the Degree of Root Resorption (mm) Between before and after treatment for the Patients in Group I (Self-ligating Brackets)

Measurements, mm	T1		T2		T2-T1	95 % CIs		*P*
	Mean	SD	Mean	SD		lower	upper	
Maxillary central incisor root	11.5	1.3	11.2	1.2	−0.3	0.17	0.43	*
Maxillary lateral incisor root	10.1	0.9	9.8	1.1	−0.2	0.04	0.56	*
Mandibular central incisor root	10.7	1.4	10.3	1.2	−0.4	0.14	0.66	*
Mandibular lateral incisor root	11.5	1.6	11.2	1.5	−0.3	0.17	0.43	*

*Statistically significant difference (*P* <05)

The same occurred for Group II, in which all of the teeth had statistically significant root resorption, (Table 3).

**Table 3:** Comparison of the Degree of Root Resorption (mm) Between before and after treatment for the Patients in Group II (Conventional Preadjusted Brackets)

Measurements, mm	T1		T2		T2-T1	95 % CIs		*P*
	Mean	SD	Mean	SD		lower	upper	
Maxillary central incisor	12.1	1.5	11.6	1.7	−0.5	0.24	0.76	*
Maxillary lateral incisor	11.5	1.7	11.2	1.5	−0.3	0.04	0.56	*
Mandibular central incisor	11.3	1.8	10.9	1.6	−0.4	0.14	0.66	*
Mandibular lateral incisor	10.9	1.8	10.6	1.9	−0.3	0.17	0.43	*

*Statistically significant difference (*P* <05)

Although the length of root resorption in Group 1 less decrease than Group 2, there was no statistically significant difference was found in the comparison of the degree of root resorption between the two groups (Table 4).

**Table 4:** Root Resorption in percentage of root shortening and in millimeters Between Group I (Self-ligating Brackets) and Group II (Conventional Preadjusted Brackets)

Measurements, mm	Group 1		Group2		95 % CIs		*P*
	Mean	SD	Mean	SD	lower	upper	
Maxillary central incisor %	2.61		4.13				NS
mm	0.3	0.4	0.5	0.3	−0.29	1.08	
Maxillary lateral incisor %	2.00		2.60				NS
mm	0.2	0.3	0.3	0.5	−0.53	1.03	
Mandibular central incisor %	3.73		3.47				NS
mm	0.4	0.4	0.4	0.5	−0.38	1.18	
Mandibular lateral incisor %	2.60		2.75				NS
mm	0.3	0.3	0.3	0.5	−0.48	1.08	

NS mean: No Statistically significant difference (*P* >05)

The distribution of teeth in the groups, scored according to Malmgren *et al.* [22], showed that Group 1 had 67.14 % of the teeth classified with scores of 0 and 1 and the remaining 32.86 % had root resorption scores of 2, 3, and 4. Group 2 had 55.71 % of the teeth with scores of 0 and 1 and 44.29 % with scores of 2, 3, and 4 (Table 5).

**Table 5:** Distribution of teeth with apical root resorption according to the scoring system of Malmgren *et al.* (1982)

Score	Group1 (*n* = 280)		Group2 (*n* = 280)		Total (*n* = 560)
	n	%	N	%	
0	0	0	0	0	0
1	188	67.14	156	55.71	344
2	67	23.93	70	25.00	137
3	22	7.86	48	17.14	70
4	3	1.07	6	2.14	9

## Discussion

Apical root resorption is a frequent undesirable side effect in orthodontic treatment. However, innovations in techniques and orthodontic materials have been developed to reduce this problem [22]. Because self-ligating brackets have the advantage of having less friction, we wanted to test the hypothesis that an association exists between self-ligating and conventional pre-adjusted brackets in amount of resorption.

Periapical radio-graphs were used to assess OIRR in the present study. This technique is in keeping with previous studies [7, 23]. It is accepted that periapical views may be insensitive to very minor changes in root lengths and may be less accurate than CBCT in studying the severity of OIRR. However,owing to the higher radiation dose applied, especially to children and adolescents when using cone beam computed tomography (CBCT) imaging, this technique requires a clear indication and benefit for the patient strictly adhering to ALARA (as low as reasonably achievable) principles in medicine [24]. It is, therefore, reasonable to infer that the use of repeated periapical films resulted in a valid assessment of incisor length in the present investigation.

Subjective methods, such as that of Malmgren et al. [21], are predominantly used in root resorption studies performed after tooth movement, presenting a primary advantage. Therefore, the subjective method used in the present seems to be reliable, showing almost perfect intra-examiner agreement and confirming the precision of the evaluation. Additionally, there were no significant systematic errors and the casual errors were within acceptable levels.

Factors related to orthodontic treatment are primarily responsible for the prevalence of root resorption [20, 25–26]. However, studies differ significantly regarding design, methodology, control group, and treatment characteristics. A small sample size is common problems that can lead to questionable results [27]. Such as Vanessa Leite [28] find that EARR has occurred in all teeth evaluated, the bracket design (self-ligating or conventional) did not demonstrate any influence on the results observed. Nevertheless, the sample only includes 19. Additionally, several studies do not distinguish the variables related to patients and treatments [26]. Although most correlated resorption with different types of techniques [20], they did not identify specific crowding. In this retrospective study, root resorption was investigated in a homogeneous sample, treated with self-ligating and conventional pre-adjusted brackets in severe anterior crowding of Class I patients.

Regarding the amount of root resorption, an average of 0.3 mm in self-ligating group and an average of 0.35 mm in conventional bracket group were found—a value close to that in the literature of 0.25 mm [21]. Although it occurred in all teeth, this degree of EARR is small and clinically irrelevant [29].

The main explanation may be due to treatment duration and light forces. Treatment duration had a positive association with the amount of EARR and has been attributed to persistent bone turnover associated with extended tooth movement. In this study, the mean treatment times were similar between the groups, it is reasonable to assume that the factors affecting treatment duration were similar and did not contribute to the difference in root resorption.

Therefore the contributory factor is not only treatment duration, but also another factor that may explain the smaller resorptions in this study to be the use of self-ligating brackets, which offer less force in relation to level displacement of teeth, thus affecting the magnitude of the EARR. Light forces have long been recommended to reduce adverse tissue reactions (root resorption) [30, 31].

Concerning the type of appliance, no statistically significant difference was found in the root resorption between conventional and Damon 3 bracket, although a trend indicating more EARR for conventional bracket was evident, but it did not reach significance. However, in the general bio-medical literature, significance levels slightly higher than the self-ligating brackets might receive critical importance depending on the severity of the health hazard imposed on the sample [32–34]. Thus, although our results should not give rise to statements on the relative capacity of specific appliance type to cause resorption, more research is needed before a definitive conclusion can be drawn on this subject.

In a relevant study comparing conventional and self-ligating brackets, no difference was found in the amount of root resorption [20]. These data are in accordance with the results of this research in which no statistically significant differences were detected between the two groups. Other studies reported the same findings [35, 36]. This implies that, although the Damon 3 bracket delivers a constant light force, this force is not of sufficient magnitude to decrease the root resorption observed after orthodontic treatment.

The results of the present study have to be interpreted with caution because of its limitations. First, this study was designed as a longitudinal retrospective study. Generally speaking, it is very difficult to avoid confounding factors. But, we can reduce the effects of bias by making strict inclusion criteria. The baseline levels of two groups are in consistency. And malocclusion type might affect the results, therefore, in this study only patients with Angle Class I were included so as to minimize the impact of confounding factors on the experimental results. Another problem related to longitudinal retrospective studies might be the information bias. We have gone through repeated trials, and surveyors and final statistics do not know the group of data. Therefore, this study is relatively real, which may reflect the actual results accurately. Second, our investigation was only assessed EARR on the incisors. Although these teeth show a higher prevalence of EARR, conclusions on EARR involvement in orthodontic tooth movement with different appliances would require examination of the entire dentition.

According to the results of this study, self-ligating brackets induced less apical root shortening measured in periapical radio-graphs than conventional pre-adjusted brackets. However, there is no statistically significant difference was found in the comparison of the degree of root resorption between the two groups. Because of self-ligatingbrackets are more expensive than conventional pre-adjusted brackets and Other advantages of this system are fewer appointments, such as improved oral hygiene, better acceptance by patients, and better treatment results, therefore choose types of brackets still depend on orthodontics experience and patients.

## Conclusion

- There are no significant differences in the amount of apical root resorption between passive self-ligating and conventional preadjusted in severe anterior crowding Class I patients.
